# Subjective well-being, general self-efficacy and coping with stress in former psychiatric patients preparing for the peer support role: an exploratory study

**DOI:** 10.1186/s12955-020-01348-6

**Published:** 2020-04-10

**Authors:** Agata Chudzicka-Czupała, Karolina Zalewska-Łunkiewicz

**Affiliations:** 1 SWPS University of Social Sciences and Humanities, Faculty of Psychology in Katowice, Department of Social and Organizational Behavior Psychology, ul. Techników 9, 40-326 Katowice, Poland; 2 SWPS University of Social Sciences and Humanities, Faculty of Psychology in Katowice, Department of Clinical and Health Psychology, ul. Techników 9, 40-326 Katowice, Poland

**Keywords:** Peer workers, Subjective well-being, Self-efficacy, Coping with stress, Psychiatric patients

## Abstract

**Background:**

People who experienced a mental crisis are involved in providing care for others who face psychiatric hospitalization. The idea of peer workforce has been developed mostly in American and European behavioral health systems. Similar program is implemented to Polish mental health care. The purpose of the study was to find out if candidates for peer support workers with different levels of subjective well-being differed also in terms of general self-efficacy and in the ways of coping with stress.

**Methods:**

As the problem has not been studied before exploratory study was conducted. The study covered a group of 72 subjects, 46 women and 26 men, aged 21–62 years (M = 41.43; SD = 10.37), former psychiatric patients, preparing for a peer worker role. We used the following questionnaires: Ryff’s Psychological Well-Being (PWB) Scales, in the Polish adaptation by Krok, the General Self-Efficacy Scale (GSES) by Schwarzer and Jerusalem, in adaptation by Schwarzer, Jerusalem and Juczyński and Brief-COPE by Carver, in adaptation by Juczyński and Ogińska-Bulik.

**Results:**

The results of cluster analysis pointed to the existence of two groups of individuals with significantly different levels of subjective well-being. Then MANOVA was used. It was determined that individuals with a higher level of well-being were characterized by a higher level of self-efficacy, a higher tendency to use positive reframing strategy and propensity towards active behavior when coping with stress, as well as by a lower propensity towards self-blaming and behavioral disengagement.

**Conclusions:**

The study demonstrates that further empirical explorations are justified. The results also encourage a search for some more possible conditions of well-being. It would be advisable to train candidates for mental health peer workers by focusing on the strengthening of their subjective well being and developing active forms of coping with stress.

## Background

Over the last few decades, firm steps have been taken across the USA and European countries towards the deinstitutionalization of psychiatric health care [1, 2]. There is a broad consensus on the need to move away from treatment in large psychiatric hospitals towards comprehensive community-based care [3, 4]. In Poland, preparations are also underway to change the psychiatric care system [5]. In 2018, community-based Mental Health Centers were launched on a pilot basis and preparations began to sanction the profession of peer worker (PW). The aim of the training program is to build the PW’ abilities in providing support and educating others with regard to healthy lifestyle or mental hygiene and thus to help them to lead meaningful lives in the community. Similar to countries such as the USA [1, 6], Germany, UK, Netherlands, Norway, Slovenia, Sweden [7], Canada, Australia, New Zealand [8] “Experienced Involvement” (EX-IN, peer counselling) programs are predicated on the notion that people with similar experiences can support each other in situations of emotional stress. It was Harry Stack Sullivan who first recruited people recovering from schizophrenia to become his assistants at the clinic for people affected by schizophrenia and nowadays the development of peer supporting in mental health services has spread to Asia, South America and Africa [9]. Although peer support is usually provided independent of conventional mental healthcare, the idea in Poland is that candidates for PWs, after taking the training, would be formally offered a job and become a part of the health system structure.

Pursuing a PW role is in line with the assumptions of empowerment, as it makes it possible to strengthen the supporter’s resources and to increase the psychological benefits of the individual receiving the help [10, 11]. The values attributed to PW support include a relationship based on a balance of power, mutual identification, a comprehensive understanding of the mental crisis, with a focus on the potential positive aspects of the situation, building a safe and trust-inspiring interpersonal relationship between the supporter and the supported individual, based on shared experience [7].

The study presented in this paper focuses on subjective well-being (SWB), and on its possible correlates, in former psychiatric patients preparing for a role. PWs use their own experience of overcoming distress and their own abilities to support others who are currently struggling with crisis. The reason for such a study results from the perspective that the EX-IN training for PWs includes self-developmental aspects which should influence their capacity to provide support and to raise their quality of life. Assessing level of SWB and its correlates among participants of PW training may be helpful for designing further work-in-group programs for strengthening particular personal competencies in using psychological resources.

Subjective well-being *is defined as “a person’s cognitive and affective evaluation of his or her life”* [12]*.* This is a subjective state of general satisfaction with life [13], associated with a sense of happiness and self-fulfillment [14], making it possible to adapt in an optimum way to changing life conditions [15]. Ryff [16, 17] proposes an eudemonistic approach to SWB, which can be described in several dimensions. The SWB of peer supporters who, having experienced a mental crisis and the resulting difficulties, intend to help other people professionally, is particularly important.

Research into the determinants of SWB variability over the course of life has not yielded unambiguous results. According to longitudinal studies [18], important life events (and a stay in a psychiatric ward may be considered as such) determine a similar percentage of variance in subjective well-being as intra-personal determinants. Extent of psychopathological symptoms was found to be inversely related to life quality and some authors suggest that SWB may not necessarily be inferred from objective life circumstances [18, 19]. According to Pavot and Diener [13], life events have a rather short-term impact on SWB because people have an ability to adapt to new life conditions which is regulated to a greater extent by temperamental and personality-related factors.

Equally important may be other psychological resources influencing well-being, described by Taylor and Broffman [20], which become particularly significant when individuals face challenges [21, 22]. Literature review, especially the content of some theories of optimal human functioning, e.g. social-cognitive theory [23, 24] or self-determination theory [25] which underline the importance of a sense of agency when dealing with life problems and an analysis of reports so far on the determinants of positive re-adaptation of individuals discharged from inpatient psychiatric treatment [26] led us to identify a variable which might correspond to important psychological resources for maintaining a high level of SWB in individuals preparing for a PW role. This variable is perceived self-efficacy, described as an important resource factor in stress appraisal process.

Perceived self-efficacy, understood as an individual’s conviction concerning their personal ability to meet diverse conditions of the specific task, leading to the achievement of the intended results [27], reflects optimistic self-confidence and the belief that one can effectively cope with adversities in various areas of social functioning [28]. People with low self-efficacy have a tendency to stress out easily and doubt their own potentials compared to those with high self-efficacy level [29]. Some studies found that general self-efficacy beliefs are predictors of mastery [30] and a determinant of SWB or a factor related with or a mediator between SWB and general health [31]. It was found that mental health problems were associated with a lack of confidence which limited everyday activities and affected opportunities both in employment and relationships [32]. Therefore, perceived self-efficacy, which is connected to setting goals for oneself and rising from failure, can be considered both as a significant psychological resource and as a factor of positive mental resilience.

Additionally, adopting stress management strategies may be significant for SWB, especially in the case of strategies focused on task-based approaches to the problem. The situation of mental crisis and psychiatric hospitalization is a very stressful, difficult and complex experience that reduces quality of life [33, 34]. Isolation from one’s natural environment, strict discipline and lack of intimacy cause a complete change in the life perspective of an individual staying in hospital, leading to stress [35]. Therefore, a mental crisis and the related hospital treatment may directly compromise the individual’s SWB, just like the post-hospitalization period, which is a time of particular risk of adaptation difficulties [36]. Exposure to the stigma of mental illness is a significant impediment [37]. Studies have shown a correlation between emotional reactions and the stress related to the stigma caused by psychiatric hospitalization with quality of life and self-esteem [38]. The manner in which psychiatric hospital patients manage to return to participation in day-to-day social life may determine their recovery process and potential improvement in SWB. The choice of an appropriate strategy for coping with stress by candidates for a PW role is also important because they will become role models for the individuals they support.

Although the well-being of psychiatric hospital patients has been given research attention [39], it has not been studied in individuals preparing for a PW role. The study presented in this paper aims to fill the gap. It is one of the first studies among individuals who decided to take on a PW role and the very first study of its kind in Poland. Its purpose was to examine whether the participants of a PW workshop differed in terms of SWB and, if so, the question was asked whether those who differed in the level of SWB also differed in level of perceived self-efficacy, in terms of the strategies for coping with stress which they adopted and in sociodemographic and medical data.

## Methods

### Study design and procedure

As the problem has not been studied before exploratory research was conducted. Purposive sampling was used. The study covered participants of the “New Expert” workshops preparing them for a peer worker role, organized by the “Leonardo” Foundation for Social Development in Krakow. These were the first workshops of this kind in Poland organized before the introduction of new legal regulations within the framework of changes in the psychiatric care system. Therefore, the study continuation will be possible after further PW trainings are launched. To avoid the potential effect of completing a workshop on what was measured the participants took part in the study during the first week of the training.

The respondents did not receive any remuneration for their participation in the survey and filled out a set of questionnaires using the paper-and-pencil method. All of them were interviewed by one of the researchers face to face in a meeting room after the workshop they participated in. To respond to the research questions quantitative methods were used. Completed questionnaires were carefully checked and the data were input after the interviews.

### Study sample

The final study sample covered 72 adults who had experienced mental health problems and at least one hospitalization in a 24/7 psychiatric ward. Some participants were excluded from the study because they did not complete all the questionnaires (*N* = 8).

### Measures

To measure *subjective well-being*, Ryff’s Psychological Well-Being Scales [16, 17], in the Polish adaptation by Krok [40],were used. The tool named in Polish “Kwestionariusz Dobrostanu Psychologicznego” (KDP) consists of 42 items on which the respondent takes a position using a Likert scale from 1 – “Strongly disagree” to 7 – “Strongly agree”. The questionnaire is composed of six scales measuring well-being in six dimensions: autonomy, environmental mastery, personal growth, positive relations with others, purpose in life, and self-acceptance. The psychometric properties of the questionnaire are satisfactory: Cronbach’s alpha for the individual scales ranges from 0.72 to 0.86.

The General Self-Efficacy Scale (GSES) [41] was used in the Polish adaptation by Schwarzer, Jerusalem and Juczyński [42] to determine the value of a one-factor *general perceived self-efficacy*. The method measures the strength of an individual’s general conviction that they can effectively cope with difficult situations and obstacles. The GSES consists of 10 statements, which are all part of one factor, on which the respondent takes a position by marking the selected answer on a Likert scale from 1 “No” to 4 “Yes”. The total score provides a general index of perceived self-efficacy. The scale has good psychometric properties – Cronbach’s alpha is 0.85.

The Brief-COPE questionnaire [43] in the Polish adaptation (Mini-COPE) by Juczyński and Ogińska-Bulik [44] was used to measure *dispositional coping with stress*, i.e. to assess the typical ways of reacting and feeling in strongly stressful situations. The tool consists of 28 statements on which the respondents take a position by marking on a Likert scale how frequently they behave in the manner described by the given statement (from 0–“I almost never do this” to 4–“I almost always do this”). Each scale comprises 2 statements. This results in a total of 14 scales measuring the frequency of adopting the following coping strategies: Active coping, Planning, Using emotional support, Using instrumental support, Venting, Behavioral disengagement, Self-distraction, Self-blame, Positive reframing, Humor, Denial, Acceptance, Religion, Substance use. The psychometric parameters obtained are satisfactory, split-half reliability is 0.86, the Guttman index 0.87.

Additionally, the respondents were asked to provide *sociodemographic data* such as age, gender, education, employment, marital status, living status and *medical data* such as number of hospitalizations, diagnosed disorder and subjective evaluation of mental health.

The measures used in the study are well theoretically grounded tools with good psychometric properties. The application of internationally known measures makes it possible to replicate the findings in different countries and to make transcultural comparisons.

### Ethical considerations

The study was conducted with due regard for ethical aspects, according to the principles of the Declaration of Helsinki and its amendments and to procedure approved by the Research Ethics Committee at the SWPS University of Social Sciences and Humanities in Katowice. It ensured anonymity of the respondents. All the individual respondents agreed to participate voluntarily, they were informed about the aim of the study and they all filled out an informed consent form.

### Statistical analysis

In order to find an answer to the question concerning the differences between the respondents in terms of SWB, k-mean cluster analysis was used. Since SWB is a multidimensional variable we used the cluster analysis as the best statistical tool that aggregates all the dimensions.

Subsequently, in order to find an answer to the questions about the differences in terms of the other variables, the MANOVA method, the Chi-square and the Student’s t tests for independent groups were used to analyze the significance of the differences between the mean results of the said variables obtained by the respondents belonging to the clusters identified.

## Results

### Demographic and medical profile of participants

The demographic and medical profile of participants was taken and can be seen in Table 1.

**Table 1:** Demographic and Medical Profile of Participants

Characteristic	Representation			
Age	Range	21–62 years		
	Mean	41.43	SD = 10.37	
Gender	Female	46 (63.89%)		
	Male	26 (36.11%)		
Educational status	Higher education	35 (48.61%)		
	Secondary education	34 (47.22%)		
	Vocational education	3 (4.17%)		
Job	Worked	34 (47.22%)	Permanently employed	19 (26.39%)
			Odd jobs	15 (20.83%)
	Unemployed	32 (44.44%)		
	Students	6 (8.33%)		
Relationships	Married or in civil unions	36 (50.00%)		
	Single	36 (50.00%)		
Living status	With a family member or partner	63 (87.50%)		
	Alone	9 (12.50%)		
Number of hospitalizations	Range	1–15		
	Mean	3.68	SD = 3.86	
Diagnosed disorder^a^	Organic disorders	4 (5.56%)		
	Schizophrenia	33 (45.83%)		
	Mood disorders	34 (47.22%)		
	Personality disorders	1 (1.39%)		
Subjective evaluation of mental health	Bad	0 (0.00%)		
	Unsatisfied	6 (8.33%)		
	Moderate	31 (43.05%)		
	Good	26 (36.12%)		
	Very good	9 (12.50%)		

*SD* standard deviation; *N* = 72

^a^Subjects were diagnosed by psychiatrists with use of the ICD-10 categories

### Descriptive statistics, variable correlations

The descriptive statistics of the studied variables (means obtained, standard deviations) and the intercorrelations between the studied variables are presented in Table 2.

**Table 2:** Descriptive statistics and correlations between the examined variables

Variable	Mean	SD	1^a^	2	3	4	5	6	7	8	9	10	11	12	13	14	15	16	17	18	19	20	21
1	33,42	6,16	1,00	0,32*	0,39*	0,32*	0,44*	0,42*	0,22	0,12	0,11	− 0,10	−0,10	− 0,14	− 0,14	0,03	− 0,21	0,02	− 0,08	− 0,05	0,08	− 0,05	− 0,07
2	32,40	7,68		1,00	0,39*	0,51*	0,49*	0,65*	0,72*	0,43*	0,34*	0,09	0,18	0,16	−0,32*	− 0,03	− 0,35*	0,45*	0,37*	0,01	0,28	0,10	0,08
3	36,85	6,02			1,00	0,40*	0,53*	0,45*	0,35*	0,39*	0,25	0,37*	0,32*	0,24	−0,28	0,12	−0,22	0,30	0,09	−0,04	0,21	−0,14	0,22
4	36,18	7,40				1,00	0,32*	0,42*	0,35*	0,36*	0,29	0,36*	0,36*	0,32*	−0,15	0,19	−0,11	0,39*	0,39*	0,08	0,36*	0,12	0,19
5	34,74	8,08					1,00	0,29	0,29	0,38*	0,28	0,18	0,17	0,02	−0,35*	−0,06	−0,22	0,16	0,06	−0,12	−0,00	0,14	0,11
6	31,64	7,96						1,00	0,52*	0,34*	0,33*	0,16	0,22	0,09	−0,29	−0,15	−0,50*	0,47*	0,10	−0,03	0,38*	0,10	−0,07
7	28,31	6,80							1,00	0,38*	0,31*	−0,09	−0,00	0,19	−0,34*	− 0,02	−0,30*	0,41*	0,31*	−0,18	0,34*	−0,08	0,07
8	2,16	0,83								1,00	0,83*	0,40*	0,52*	0,50*	−0,02	0,37*	0,14	0,57*	0,51*	0,22	0,60*	0,29	0,39
9	2,15	0,88									1,00	0,35*	0,44*	0,46*	-0,12	0,24	0,17	0,57*	0,47*	0,11	0,61*	0,22	0,37
10	2,18	0,98										1,00	0,90*	0,65*	0,25	0,33*	0,22	0,49*	0,39*	0,36*	0,48*	0,33*	0,46
11	2,22	0,96											1,00	0,68*	0,19	0,39*	0,17	0,57*	0,51*	0,39*	0,55*	0,43*	0,39
12	1,92	0,83												1,00	0,27	0,52*	0,32*	0,52*	0,62*	0,33*	0,50*	0,39*	0,46
13	1,19	0,88													1,00	0,57*	0,53*	0,15	0,29	0,61*	0,20	0,29	0,46
14	2,09	0,79														1,00	0,45*	0,44*	0,61*	0,58*	0,46*	0,38*	0,48
15	1,69	0,87															1,00	0,03	0,12	0,20	0,11	0,18	0,36
16	2,22	0,82																1,00	0,66*	0,41*	0,77*	0,26	0,45
17	1,48	0,71																	1,00	0,44*	0,60*	0,43*	0,51
18	1,17	0,74																		1,00	0,23	0,20	0,49
19	2,31	0,88																			1,00	0,28	0,37
20	1,65	0,97																				1,00	0,16
21	0,88	0,68																					1,00

*p < 0.01; SD - standard deviation; *N* = 72

^a^The numbers indicate variables as follow: 1-Autonomy; 2-Environmental mastery; 3-Personal growth; 4-Positive relations; 5-Purpose in life; 6-Self-acceptance; 7-General self-Efficacy; 8-Active coping; 9-Planning; 10-Using emotional support; 11-Using instrumental support; 12-Venting; 13-Behavioural disengagement; 14-Self-distraction; 15-Self-blame; 16-Positive reframing; 17-Humour; 18-Denial; 19-Acceptance; 20-Religion; 21-Substance use

### Subjective well-being – differences between respondents

The k-mean clustering method maximizes the distance between the isolated clusters, and their number is not known a priori. The STATISTICA package includes v-fold cross-validation, which is an algorithm for the automatic determination of the optimal number of clusters. The number of clusters isolated is 2 and corresponds to two groups of respondents, differing to the maximum extent in terms of the individual dimensions of SWB.

**Fig. 1:**
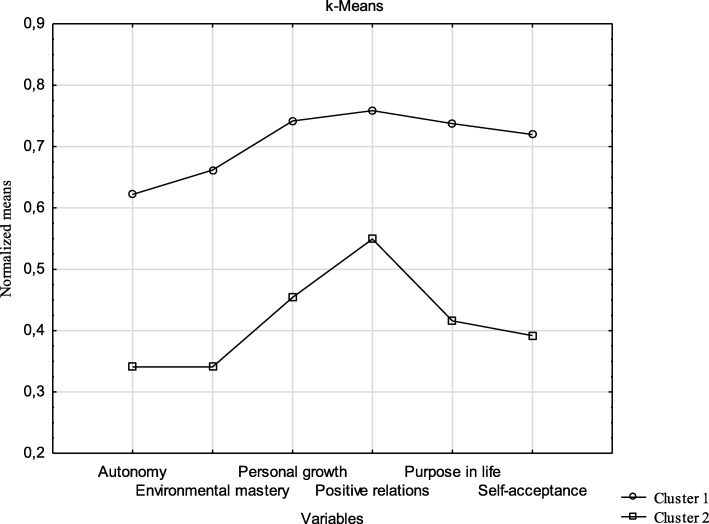
Graph of variables means

Table 3 presents the mean values of the individual variables (SWB dimensions) corresponding to the isolated clusters of respondents and the results of variance analysis of the differences between these mean values. The mean values for all four clusters are presented in the graph (Fig. 1).

**Table 3:** Centroids for k-means clustering and ANOVA variance analysis for the variables creating the clusters

Variables	Mean		Between SS	*df*	Within SS	*df*	*F*^*c*^	*p*
	Cluster 1^a^	Cluster 2^b^						
Autonomy	37.17	29.86	960,20	1	1731,30	70	38,82	0.00
Environmental mastery	37.51	27.57	1779,50	1	2409,82	70	51,69	0.00
Personal growth	40.54	33.35	930,20	1	1641,12	70	39,68	0.00
Positive relations	39.51	33.03	756,94	1	3133,72	70	16,91	0.00
Purpose in life	39.86	29.89	1786,13	1	2843,85	70	43,96	0.00
Self-acceptance	37.03	26.54	1978,45	1	2516,16	70	55,04	0.00

^a^N1 = 35; ^b^N2 = 37; ^c^F – univariate test

The results demonstrate that the respondents from both clusters that were selected differ significantly in terms of all SWB dimensions, i.e. in terms of autonomy, environmental mastery, personal growth, positive relations with others, purpose in life, and self-acceptance. Individuals belonging to cluster 1 score significantly higher on all SWB scales than those from cluster 2.

### Perceived self-efficacy, strategies for coping with stress, demographic and medical characteristics–analysis of differences

The results of the comparison between individuals from both clusters in terms of perceived self-efficacy and the strategies for coping with stress the respondents adopted were based on an analysis of the differences between mean values, carried out using the MANOVA method. The results are shown in Table 4.

**Table 4:** MANOVA Variance Analysis, independent variable: cluster

All effects (Wilks’ test): F = 2.32 *p* = 0.01** Partialeta-squared =0.38			
Dependent variables	Mean		F^a^
	Cluster 1.	Cluster 2.	
General self-efficacy	30.89	25.86	11.21**
Active coping	2.44	1.89	8.80**
Planning	2.34	1.97	3.26
Using emotional support	2.31	2.05	1.26
Using instrumental support	2.34	2.10	1.21
Venting	1.96	1.88	0.16
Behavioural disengagement	0.93	1.43	6.37**
Self-distraction	2.07	2.11	0.04
Self-blame	1.37	1.99	10.26**
Positive reframing	2.47	1.99	6.85**
Humour	1.54	1.42	0.55
Denial	1.19	1.16	0.02
Acceptance	2.49	2.14	2.94
Religion	1.57	1.72	0.40
Substance use	089	0.87	0.02

* *p* < 0.05; ** *p* < 0.01; ^a^F – univariate test

The fact of belonging to the clusters that were identified explains 38% of the variance in terms of the respondents’ SWB, while the multidimensional test for all the effects (F = 2.32, for *p* = 0.01) indicates that the model is statistically significant. Therefore, it should be assumed that there are statistically significant differences between the clusters of respondents that were identified in terms of all the dependent variables taken together.

The analysis shown in Table 4 of the one-dimensional differences with regard to the individual variables between the two clusters identified proves that there are, however, statistically significant differences between individuals from cluster 1 and cluster 2 which concern five dependent variables. Respondents from cluster 1, with a significantly higher degree of well-being than those from cluster 2, also score significantly higher in terms of perceived self-efficacy. The individuals from cluster 1 also apply the active coping and positive reframing significantly more frequently, while self-blame and behavioral disengagement strategies less frequently.

The analysis of the differences between the clusters in relation to demographic and medical characteristics, conducted using the Chi-square test and the Student’s t test for independent groups, indicated that among all the variables included in Table 1, statistically significant differences concern only three traits: sex (χ2=10.62, *p* = 0.00), education (t = 2.9, *p* = 0.00) and work (t = 2.03, *p* = 0.04). Cluster 1 has a much higher proportion of women, people with higher education, and people with permanent or temporary jobs than those selected in cluster 2.

## Discussion

The results made it possible to distinguish, among the PW workshop participants who were studied, two clusters of individuals differing significantly in terms of the SWB level. The existence of significant differences was also confirmed between individuals from the groups thus distinguished in terms of psychological resources, i.e. traits selected because of their potential significance for SWB, such as perceived self-efficacy and a tendency to apply strategies for coping with stress relying on active coping, positive reframing, behavioral disengagement and self-blame.

Referring to the results of the study on SWB conducted by Ryff [16] and to her descriptions of the SWB dimensions, it can be concluded that the respondents from cluster 1 are more autonomous and self-directed and they yield to outside pressure to a smaller extent than those belonging to cluster 2. They also judge themselves through personal standards, rather than depending on societal standards. They have a greater sense of environmental mastery, awareness of their own competences and a greater ability to transform the environment in accordance with values that are important to them, so they may find it easier to cope with the requirements coming from the environment. Individuals from this cluster also have a greater ability to fulfill their potential and to improve their skills compared to those from cluster 2. They also have more positive relations with others and they are more capable of trusting others and making friends compared to those belonging to cluster 2. They find it easier to control their own lives, pursue their goals, and find meaning in what they do. Respondents from cluster 1 accept themselves more, pursue self-fulfillment to a greater extent, and may also find it easier to accept their own flaws and see the advantages, which favors mental health, strengthens a realistic view of oneself, and that may give them greater resistance to stigma.

The results indicate that the individuals from the cluster scoring significantly higher on the Subjective Well-Being Scale also have significantly higher self-efficacy scores than the individuals from the other cluster. This continues to be in line with previous studies pointing to positive correlations between SWB and perceived self-efficacy [45]. Effective control of one’s own actions and achievement of goals, using active coping and planning strategies, connected with self-efficacy [27], may be an important element of recovery and of internal increasing of mental strength, and thus of a sense of general satisfaction, which is of key importance in the concept of SWB, and an especially significant one for future peer supporter staff, as agency is immanent to the PW role.

It seems that the presence of dispositions and traits such as activity, tendency to positive reframing, self-confidence, self-efficacy and satisfaction with one’s actions is also desirable in the PW role, given that providing professional help requires confidence in one’s own resources and frequent initiation of actions. According to the results, individuals from the cluster scoring higher on the SWB scale also adopt the strategy for coping with stress consisting in behavioral disengagement and self-blame much less frequently.

Higher resourcefulness of subjects from cluster 1 seem to be proved also by other differences between them and subjects from cluster 2: the significantly higher number of persons with higher education and with a job in cluster 1 may also suggest that they have other resources which respondents belonging to cluster 2 may be deprived of. There is also a significantly higher proportion of women in the cluster 1 than in the cluster 2, which may be important. With such a small number of respondents, however, cautious conclusions should be drawn regarding this result.

### Limitations of the study

The research has its limitations. The small number of respondents is certainly one of them. However, this was determined by the specificity and unique nature of the sample. The respondents were participants of the one of the first workshop in Poland organized for PWs, so their number was determined by the number of the course participants. Considering the fact that the research was of an exploratory nature, in order to verify the initial observations made here, it is planned to continue the investigations with a much larger sample. This will only be possible in the future once participants of further training courses have been recruited, after the new legislation is enacted. The study does not make it possible either to define the determinants of the differences observed in the level of SWB between the respondents, but the very acknowledgement of the existence of such differences may contribute to building in-depth models of dependence analyses.

### Future research directions and practical implications of the study

SWB, in eudemonistic terms, is a significant determinant of satisfactory relationships with other people, a predictor of commitment to work, a trait correlated positively with openness towards experience, the tendency to compromise and conscientiousness, and negatively with neuroticism, a trait considered both as a consequence of the earlier health condition and as a predictor of mental and physical health [16]. Taking into account the differences between the survey respondents in relation to this variable, it seems to be vital to continue research in a group of individuals preparing for a PW role in terms of their level of SWB and of the individual psychological resources strengthening SWB, as well as of the ways of coping with stress, since these traits may constitute important conditions determining success in supporting others.

The research methodology could be improved by triangulating it with qualitative methods, for example ones based on interpretative phenomenological analysis, allowing the participants to express themselves more freely when describing their feelings concerning well-being and its determinants. Other psychological resources, such as resilience, self-esteem and optimism, could also be taken into account in further studies of PW candidates.

Social support, which is an important resource as a predictor of well-being and coping with stress [46, 47], not analyzed here, may also play a key role. As there are more variables that have been demonstrated to be effective in the transfer to community life of former psychiatric patients it is difficult to mention here all of them [48].

Research implications suggest that the findings may be important for the practice. It would be advisable to train individuals preparing for a PW role by focusing on the strengthening of SWB and its personality-related correlates and developing active forms of coping with stress. Thus interventions and trainings might help promote health, improve the quality of life of former psychiatric patients and, finally, help them to support others.

## Conclusions

This preliminary analysis shows that there are statistically significant differences between candidates for PW role. The respondents differ significantly in terms of SWB. Those with higher SWB also have higher perceived self-efficacy, less often cope with stress by refraining from an attempt to resolve the difficult situation and blaming themselves, and are more determined to use positive reframing strategy, notice the optimistic aspects of the situation and actively fight. Continuation of the study will be possible after further PW trainings are launched.

## Data Availability

The datasets used and analysed during the current study are available from the corresponding author on reasonable request.

## Abbreviations

PW: Peer worker
SWB: Subjective well-being
EX-IN: Experienced involvement
